# Impact of Brachial-Ankle Pulse Wave Velocity on Myocardial Work by Non-invasive Left Ventricular Pressure-Strain in Non-hypertensive and Hypertensive Patients With Preserved Left Ventricular Ejection Fraction

**DOI:** 10.3389/fcvm.2022.814326

**Published:** 2022-02-10

**Authors:** Qin Duan, Dongying Zhang, Qian Dong, Kangla Liao, Yunjin Yang, Liu Ye, Ping Ge, Shu Qin

**Affiliations:** ^1^Department of Cardiology, The First Branch, The First Affiliated Hospital of Chongqing Medical University, Chognqing, China; ^2^Department of Cardiology, The First Affiliated Hospital of Chongqing Medical University, Chongqing, China

**Keywords:** arterial hypertension, brachial-ankle pulse wave velocity, echocardiography, myocardial work, left ventricular performance

## Abstract

**Objective:**

Data regarding the influence of arterial stiffness on myocardial work (MW) has been scarce. This study was performed to investigate the association between brachial-ankle pulse wave velocity (baPWV) and MW by non-invasive left ventricular pressure–strain in a population of non-hypertensive and hypertensive individuals.

**Methods:**

Two hundred and eight participants (104 hypertensive and 104 non-hypertensive individuals) were prospectively enrolled into the study. All participants underwent conventional echocardiography, as well as 2D speckle-tracking echocardiography to assess MW by non-invasive left ventricular pressure–strain and global longitudinal strain (GLS). baPWV measurements were made at the same day as the echocardiography. Then, participants were categorized according to baPWV tertiles. Correlation between baPWV and MW were analyzed. Predicting ability of baPWV for abnormal WM was analyzed using receiver operating characteristic (ROC) curve.

**Results:**

The median baPWV from the low to high tertile groups were 1286.5 (1197.5–1343.5), 1490.0 (1444.5–1544.0), and 1803.8(1708.3–1972.0) cm/s, respectively. In simple linear regression analysis, baPWV had a significant positive association with global work index (GWI), global constructed work (GCW), and global wasted work (GWW), and a negative association with global work efficiency (GWE). The association remained significant after adjusting for major confounding factors in multiple linear regression analysis. The areas under the ROC curve of baPWV for predicting abnormal GWI, GCW, GWW, and GWE were 0.653, 0.666, 0.725, and 0.688, respectively (all *p* < 0.05).

**Conclusions:**

BaPWV is significantly associated with all four components of MW using non-invasive left ventricular pressure-strain method in a mixed population of non-hypertensive and hypertensive individuals.

## Introduction

An accurate and very early identification of the impairment in left ventricular contractility is pivotal in terms of prognosis in a majority of cardiac disease (1). Left ventricular ejection fraction (LVEF) is the routine used index of systolic function. Recently, left ventricular global longitudinal strain (LVGLS), assessed by speckle-tracking echocardiography, is increasingly utilized to evaluate even sub-clinical alterations in left ventricular (LV) function where LVEF is normal (2, 3). However, these two methodologies are limited by their load dependency (4).

Theoretically speaking, myocardial work (MW) is a reliable index for assessment of myocardial function as it reflects the myocardial O_2_-metabolism. However, it is never implemented in clinical routine, because it is traditionally evaluated by an invasive pressure-volume loop (5). Recently, according to a similar principle, Russell et al. assessed segmental and global MW by measuring LV pressure-strain loop (LV-PSL), a non-invasive method (brachial artery cuff pressure) (6). As it took into account deformation as well as afterload, the MW by non-invasive LV-PSL potentially offered more incremental value than strain and LVEF to myocardial function assessment. Therefore, it has been applied in research regarding different clinical settings including heart failure, acute coronary syndrome, cardiac dyssynchrony, etc. (4). Furthermore, Chan et al. showed that MW assessment was beneficial for hypertensive patients, as MW index showed an increase in acute pressure overload, while EF and LVGLS remained unaffected (7).

Arterial stiffness is an important manifestation of sub-clinical organ damage and provides an established marker of cardiovascular disorders (8). It is not only an important risk factor, but a vital pathological change of hypertension (9). Brachial-ankle pulse wave velocity (baPWV), as a reliable indicator of arterial stiffness, is increasingly being considered in clinical settings (10, 11). Meta-analysis conducted in various populations has shown that the increase in baPWV is associated with an increased risk of cardiovascular events (11).

The heart and the arterial system are anatomically and functionally linked. It has been reported that arterial stiffness impairs myocardial relaxation and augments LV stiffness and end-diastolic LV pressure (12). Additionally, premature wave reflections caused by arterial stiffness can decrease pressure in diastole which further decrease coronary perfusion (13). For these reasons, it seems that there is a possible relationship between arterial stiffness and MW. Accordingly, this study was performed to investigate the association between baPWV and MW by non-invasive LV-PSL to find out whether baPWV could predict abnormal MW.

## Methods

### Study Population

**Figure 1:**
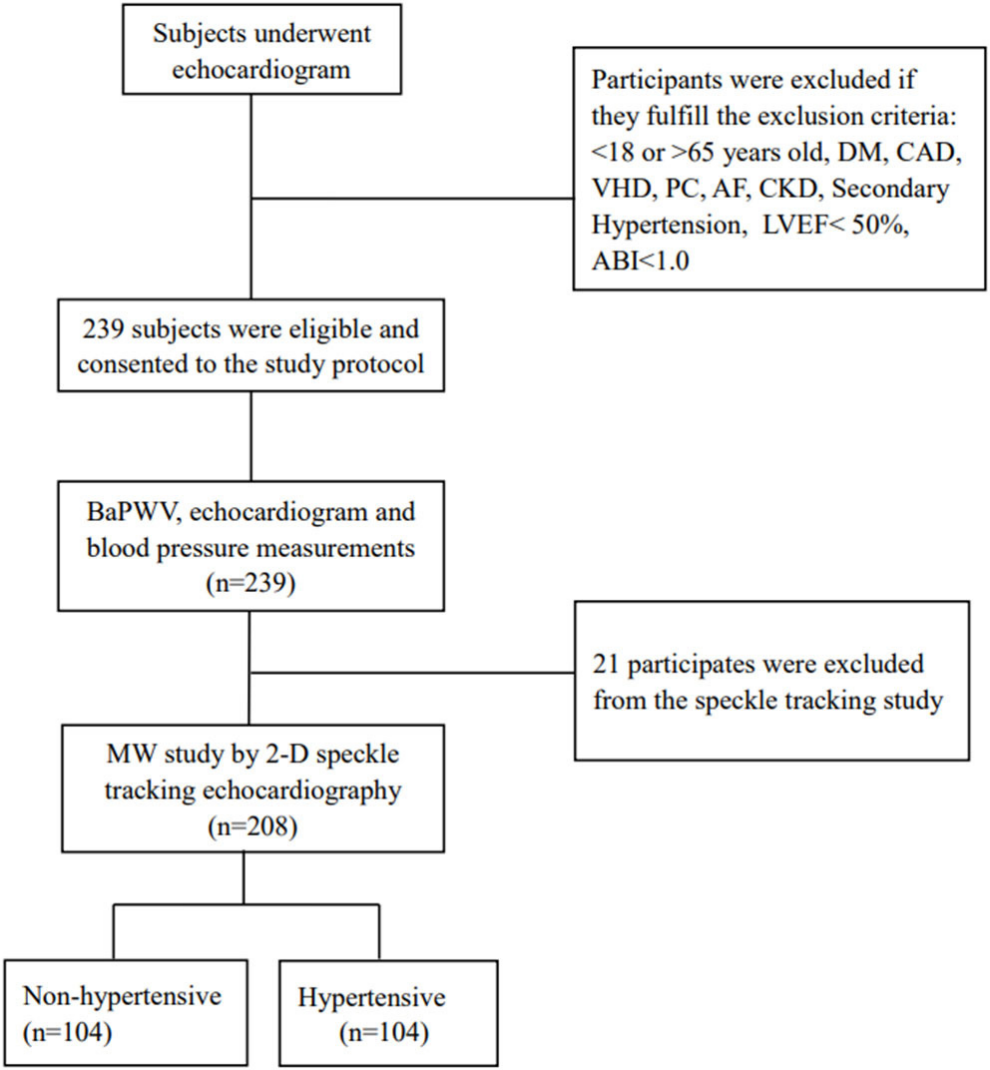
Flow diagram for the study population. ABI, ankle-brachial index; AF, arterial fibrillation; CAD, coronary artery disease; CKD, chronic kidney disease; DM, diabetes mellitus; LVEF, left ventricular ejection fraction; MW, myocardial work; PC, primary cardiomyopathies; VHD, valvular heart disease.

This is a single-center, prospective study conducted in echocardiography room of the first affiliate hospital of Chongqing Medical University from September 1, 2020 and December 30, 2020. The study included consecutive hypertensive and non-hypertensive individuals aged between 18 and 65 years old, with LVEF equal to 50% or higher. Two hundred and thirty nine subjects were evaluated via echocardiography and baPWV. Of those, 208 participants underwent 2D speckle-tracking echocardiography for assessment of MW by non-invasive LV-PSL and LVGLS. Participants with documented diabetes mellitus, coronary artery disease or suggestive symptoms, valvular heart disease, atrial fibrillation, chronic kidney dysfunction, primary cardiomyopathies, secondary causes of hypertension, and ankle-brachial index (ABI < 1.0) were excluded from the study. Twenty one subjects did not undergo speckle-tracking analysis because of inability to fit with the study devices or suboptimal echocardiographic window (Figure 1).

All participants underwent full clinical evaluation, including history of hypertension, the usage of antihypertensive drugs, status of smoking and alcohol use, height, weight, waist, blood pressure and electrocardiograph, etc. Hypertension was defined as systolic blood pressure ≥ 140 mmHg and/or diastolic blood pressure ≥ 90 mmHg with use of any anti-hypertensive medication or not (14). Current smoking was defined as smoking at least one day over the past 30 days (15). Current drinking was defined as consuming at least one alcoholic drink over the previous 30 days (16).

Brachial blood pressure in the right arm was measured using an automated digital oscillometric sphygmomanometer (Omron HEM-7312, Matsusaka, Japan) and immediately performed before the echo exam with the patient in supine position. Three sequential measurements with one minute interval were obtained, and the mean values were taken as the blood pressures for statistical analysis.

This study was registered in clinicaltrials.gov (approval No. NCT04573257). It was conducted in accordance with the “Declaration of Helsinki” and approved by the ethics committee of the first affiliated hospital of Chongqing medical university (approval No. 2020-606). Informed consents were obtained from all participants.

### Echocardiography Examination

Echocardiography was performed by 3 trained experienced sonographers using a GE E95 machine (GE, Vingmed Ultrasound, Horten, Norway) equipped with a M5Sc transthoracic transducer. All recordings and measurements were performed in line with current American Society of Echocardiography recommendations (17). All participants were examined with conventional 2D, Doppler, and speckle-tracking deformation imaging. Patients were scanned in the left lateral decubitus position. Multiple consecutive cardiac cycles of standard echocardiography 2D images were acquired and stored digitally for subsequent analysis.

Left atrial volume and LV volumes were calculated using the area-length methods and the Simpson biplane method. LV mass was calculated using the linear method and normalized by body surface area, and sex-dependent cut-off values were applied to indicate LV hypertrophy (LVH) (18).

Myocardial deformation of the LV was assessed using a semi-automated 2D speckle-tracking technique in the three apical views by one experienced sonographer blind to the other information of the participants. Images were analyzed using GE Echopac software (GE Medical Systems, version 203.88) after manual tracing of the endocardial border at the end systole of the LV. Once the aortic values closure was set, the system automatically followed the myocardial wall motion at the cardiac cycle. GLS was calculated as the average values of all the 18 segments. To be consistent with clinical meaning, the higher values the better strain deformation, absolute GLS value was used for statistical analysis. Intra-operator variability exhibited excellent consistency (correlation coefficient = 0.93, *P* < 0.001).

Left ventricular myocardial work is a novel echographic marker that reflects the stroke work of the LV and is estimated by using the brachial artery blood pressure and LVGLS (6). MW was measured from pressure-strain loops areas constructed from the LV pressure curves combined with GLS. MW and its components, namely global work index (GWI), global constructed work (GCW), global wasted work (GWW), and global work efficiency (GWE) was calculated step by step. That is, the GLS of LV was firstly estimated, followed by timing the valvar events including the opening and closure of mitral valve and aortic valve and finally recording the brachial blood pressure of the participants. The MW was calculated from the closure to the opening of the mitral valve. The area of the pressure strain loop represented the MW index. Constructive work was defined as work during shortening in systole and lengthening in isovolumetric relaxation; conversely, wasted work was the work performed during lengthening in systole and shortening in isovolumetric relaxation; and constructive work divided by the sum of constructive and wasted work served as work efficiency. The methodology above mentioned was validated in previous publications (4). On the basis of previous studies, abnormal MV was defined as GWI ≥ 2,200 mmHg%, GWE ≤ 97%, GCW ≥ 2,500 mmHg% and GWW ≥ 90 mmHg% (19–21).

### baPWV

The baPWV was measured using an automated PWV/ABI analyzer (Omron Colin BP-203RPE III). After at least 5 min of rest in the supine position, four blood pressure cuffs were wrapped around both bilateral upper arms and ankles and then connected to a plethymographic sensor and oscillometric pressure sensor. The equipment recorded the volume pulse form, phonogram, and arterial blood pressure at the bilateral brachia and ankles. The distance between the right brachium and ankle was estimated base on the subject's height. The baPWV was automatically calculated as the distance divided by the transmission time. In addition, ABI was calculated bilaterally as the ratio of ankle-SBP to brachium-SBP on the left and right sides. The mean baPWV and ABI for the left and right sides were used for analysis. baPWV measurements were made on the same day as the echocardiography.

### Statistical Analysis

The quantitative variables were expressed as mean ± standard deviation (SD) or median (interquartile range) based on the normality of their distribution evaluated by the Shapiro-Wilk test. The qualitative variables were expressed as frequencies and percentages. Analyses were carried out by categorizing the participants into tertiles according to the baPWV. Differences among the three groups were analyzed for statistical significance with variance (ANOVA) testing when comparing variables with normal distribution and Kruskal Wallis test for non-normally distributed variables. When comparing categorical data, Chi-square or the Fisher's exact test were used for comparison. Spearman correlation analysis was used for assessing the correlates of MW indexes. Univariate and multivariate linear regression analyses were performed using all potentially relevant variables to identify baseline independent predictors of 4 MW indexes including GWI, GWE, GCW, and GWW. The variables included in the regression model were: age, sex, body mass index (BMI), smoking and drinking status, hypertension, antihypertensive medication use, LVEF, cardiac index (CI), stroke volume (SV), Left atrial volume index (LAVI), E/e' Septum, Left ventricular mass index (LVMI), GLS, as well as baPWV. Only variables with statistically significant *P*-values (*p* < 0.05) in univariate analysis were included in the multivariate regression model. Potential multicollinearity was considered by computation of variance inflation factor. Collinearity was considered acceptable and the regression model was considered stable for a variance inflation factor of <3. Receiver operating characteristic (ROC) curve was used to detect the diagnostic value of baPWV for abnormal MV parameters. All analyses were performed with SPSS version 19.0 and GraphPad Prism version 7.0. *P*-values of two-tailed < 0.05 were considered statistically significant for all described analyses.

## Results

### Clinical Characteristics

The final analysis included 208 participants with 104 of them diagnosed as hypertension. The study populations were categorized into tertiles according to the baPWV. Table 1 showed the demographic and clinical data of the study population in tertile groups. The median age of the whole population was 49.0 (42.0–56.0) years, and 114 (54.8%) subjects were men. The median value of baPWV was 1491.5 (1343.3–1710.8) cm/s. LVH was detected in 57 (27.4%) of the pooled population. The median baPWV from the low to high tertile groups were 1286.5 (1197.5–1343.5), 1490.0 (1444.5–1544.0), and 1803.8 (1708.3-1972.0) cm/s, respectively. The higher baPWV tertile was proportionally associated with old age, blood pressure, waist, hypertension, and LVH. The three groups were comparable to the proportion of current smoking and drinking.

**Table 1:** Baseline clinical characteristic in the study population and in tertile groups.

**Variable**	**Overall (*n* = 208)**	**T1 (*n* = 69)**	**T2 (*n* = 69)**	**T3 (*n* = 70)**	***P***
Age (years)	49.0 (42.0–56.0)	47.0 (38.5–53.5)	47.0 (39.0–52.0)	54.5 (48.0–60.0)	<0.001
Male/Female	114/94	29/40	45/24	40/30	0.021
SBP (mmHg)	133.3 ± 19.1	118.7 ± 13.2	134.4 ± 13.6	146.8 ± 18.5	<0.001
DBP (mmHg)	83.0 (77.0–90.8)	76.0 (69.0–81.0)	85.0 (80.0–89.0)	97.5 (68.5–109.8)	<0.001
HR (bpm)	76.0 (64.8–84.0)	68.69 (62.01–80.54)	78.73 (68.81–84.42)	77.28 (66.21–86.50)	0.003
baPWV (cm/s)	1491.5 (1343.3–1710.8)	1286.5 (1197.5–1343.5)	1490.0 (1444.5–1544.0)	1803.8 (1708.3–1972.0)	<0.001
ABI	1.1 (1.1–1.2)	1.1 (1.0–1.1)	1.1 (1.1–1.2)	1.1 (1.1–1.2)	<0.001
BMI (kg/m^2^)	24.1 (21.9–28.4)	22.9 (21.3–25.7)	25.3 (22.4–26.7)	24.6 (21.9–26.8)	0.021
Waist (cm)	84.0 (78.0–91.0)	80.0 (75.0–88.5)	86.0 (81.0–93.5)	86.0 (80.0–92.0)	0.001
Current smoking	53 (25.5%)	13 (18.8%)	17 (24.6%)	23 (32.9%)	0.163
Current drinking	54 (26.0%)	16 (23.2%)	20 (29.0%)	18 (25.7%)	0.783
Hypertension	104 (50.0%)	12 (17.4%)	38 (55.1%)	54 (77.1%)	<0.001
Antihypertensive medication use	56 (26.9%)	10 (14.5%)	20 (29.0%)	26 (37.1%)	0.010
ARB	17 (8.2%)	4 (5.8%)	4 (5.8%)	9 (12.9%)	0.214
ACEI	1 (0.5%)	1 (1.4%)	0 (0.0%)	0 (0.o%)	0.363
CCB	40 (19.2%)	6 (8.7%)	16 (23.1%)	18 (25.7%)	0.021
β-block	12 (5.8%)	4 (5.8%)	3 (4.3%)	5 (7.1%)	0.779
diuretics	5 (2.4%)	2 (2.9%)	1 (1.4%)	2 (2.9%)	0.818
**α**-block	1 (0.5%)	0 (0.0%)	1 (1.4%)	0 (0.0%)	0.363
LVH in UCG	57 (27.4%)	8 (11.6%)	18 (26.1%)	31 (44.3%)	<0.001

*The cutoff values of mean baPWV for each tertile were 1401.5 and 1598.5 cm/s*.

*ARB, angiotensin receptor blockers; ACEI, angiotensin converting enzyme inhibitors; baPWV, brachial-ankle pulse wave velocity; BMI, body mass index; CCB, Calcium channel blockers; DBP, diastolic blood pressure; HR, heart rate; LVH, left ventricular hypertrophy; SBP, systolic blood pressure; UCG, ultrasound cardiogram*.

### Standard and Speckle-Tracking Echocardiographic Data

Table 2 revealed the routine echocardiographic and speckle-tracking echocardiographic parameters of the study population in tertile groups. The mean left ventricular global longitudinal strain of the whole population was 19.7 ± 2.1%. Interventricular septal wall thickness (IVST), posterior wall thickness (PWT), cardiac Index (CI), left ventricular mass index (LVMI), E/e' Septum were positively related to the baPWV tertile. Three components of MW tended to increase according to the baPWV tertile, except GWE. GWE and GLS were inversely associated to the baPWV. No differences were found between left ventricular end diastolic dimension (LVEDD), left ventricular end systolic dimension (LVESD), fractional shortening (FS), LVEF, stroke volume (SV), and left atrial volume index (LAVI) and the tertile groups.

**Table 2:** Ehocardiographic data in the study population and in tertile groups.

**Variable**	**Overall (*n* = 208)**	**T1 (*n* = 69)**	**T2 (*n* = 69)**	**T3 (*n* = 70)**	***P***
Standard echocardiographic data
IVST (mm)	10.0 (9.0–11.0)	9.0 (9.0–10.0)	10.0 (10.0–11.0)	11.0 (10.0–12.0)	<0.001
PWT (mm)	10.0 (9.0–11.0)	9.0 (8.0–10.0)	10.0 (9.0–11.0)	10.0 (9.0–12.0)	<0.001
LVEDD (mm)	46.0 (43.0–48.0)	45.0 (44.0–48.0)	46.0 (44.0–52.0)	47.0 (43.0–49.0)	0.285
LVESD (mm)	30.0 (28.0–32.0)	29.0 (27.0–31.0)	30.0 (28.0–31.5)	30.0 (28.0–32.0)	0.247
FS (%)	35.0 (33.0–37.0)	35.0 (33.0–37.0)	34.0 (33.0–36.0)	35.0 (33.0–37.0)	0.627
LVEF (%)	63.9 ± 3.4	64.0 ± 3.5	63.7 ± 3.2	63.9 ± 3.5	0.856
CI(L/min/m^2^)	2.7 (2.4–3.1)	2.6 (2.1–2.9)	2.7 (2.4–3.0)	2.8 (2.5–3.3)	0.001
SV(ml)	61.9 ± 11.6	60.2 ± 10.3	61.6 ± 10.5	64.0 ± 13.7	0.157
LVMI (g/m^2^)	93.6 (81.9–108.8)	83.1 (76.5–94.7)	95.8 (95.7–104.2)	102.4 (90.0–118.8)	<0.001
LAVI (ml/m^2^)	25.1 (21.6–31.2)	25.1 (22.1–28.6)	29.2 (24.4–37.2)	27.0 (22.8–34.1)	0.086
E/e' Septum	10.1 (8.4–12.4)	9.0 (7.3–10.6)	10.1 (8.5–12.0)	11.6 (9.8–13.6)	<0.001
Myocardial work and strain data
GWI (mmHg%)	1923.0 (1690.5–2140.5)	1745.0 (1608.0–1991.5)	1995.0 (1702.5–2154.0)	2034.5 (1769.3–2270.8)	<0.001
GWE (%)	95.0 (93.0–96.0)	96 (94.0–97.0)	95.0 (93.0–96.5)	93.0 (90.0–95.0)	<0.001
GCW (mmHg%)	2335.9 ± 348.8	2168.0 ± 259.8	2345.0 ± 337.2	2492.3 ± 365.3	<0.001
GWW (mmHg%)	102.5 (71.0–162.8)	83.0 (54.0–115.0)	89.0 (61.5–138.5)	155.0 (107.8–232.3)	<0.001
GLS (%)	19.7 ± 2.1	20.4 ± 2.2	19.7 ± 1.9	19.2 ± 2.1	0.003

*baPWV, brachial-ankle pulse wave velocity; CI, cardiac index; FS, fractional shortening; GCW, global constructed work; GLS, global longitude strain; GWE, global work efficiency; GWI, global work index; GWW, global wasted work; IVST, interventricular septal wall thickness; LAVI, left atrial volume index; LVEDD, left ventricular end-diastolic dimension; LVESD, left ventricular end-systolic dimension; LVEF, left ventricular ejection fraction; LVMI, left ventricular mass index; PWT, posterior wall thickness; SV, stroke volume*.

Baseline clinical data and ehocardiographic data in non-hypertensive and hypertensive participants were shown in Supplementary Table S1. Hypertensive patients had significantly higher baPWV as compared with that in the non-hypertensive group. Most of the echocardiographic parameters were significantly higher in the hypertensive group. Of notice, GWI, GCW, GWW, were significantly higher and GLS and GWE were significantly lower in the hypertensive group.

### Relationship Between MW Indexes and baPWV

**Figure 2:**
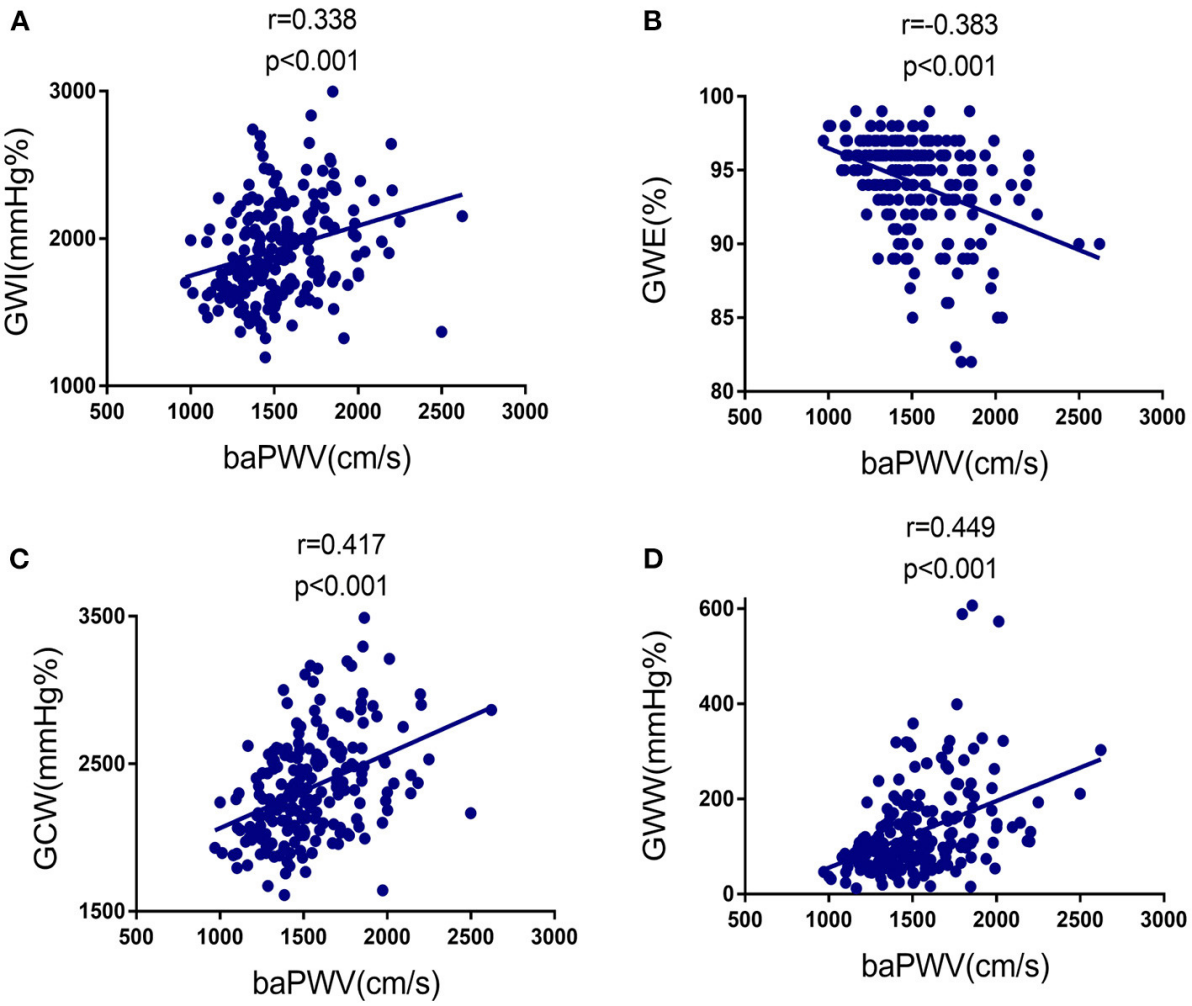
Correlation between baPWV and the four components of MW in the study population. **(A)** Relationship of baPWV to GWI. **(B)** Relationship of baPWV to GWE. **(C)** Relationship of baPWV to GCW. baPWV, brachial-ankle pulse wave velocity; GCW, global constructed work; GWE, global work efficiency; GWI, global work index; GWW, global wasted work; MW, myocardial work.

We found linear and positive associations of the baPWV with GWI (*r* = 0.338, *p* < 0.001), GCW (*r* = 0.417, *p* < 0.001), and GWW (*r* = 0.449, *p* < 0.001), as well as linear and negative association of the baPWV with GWE (*r* = −0.308, *p* < 0.001), as shown in Figures 2A–D; Table 3. Correlations between baPWV and the 4 components of MW in both non-hypertensive and hypertensive subgroups were shown in Supplementary Figure S1. Supplementary Table S2 provided simple association analyses of MW parameters and major clinical data/other echocardiographic parameters. In addition, hypertension, E/e', LVMI, and GLS were also significantly associated with GWI, GWE, GCW, and GWW.

**Table 3:** Association of baPWV with global work index, global work efficiency, global constructed work and global wasted work.

	**Univariable analysis**				**Multivariable analysis**			
	**B**	**95%CI**	**β**	***P***	**B**	**95%CI**	**β**	***P***
^a^GWI (mmHg%)	122.100	70.404–173.796	0.309	<0.001	72.933	27.769-118.098	0.186	0.002
^b^GWE (%)	−1.525	−2.036–(−1.015)	−0.380	<0.001	−0.789	−1.380–(−0.198)	−0.199	0.009
^c^GCW (mmHg%)	162.079	108.007–216.150	0.381	<0.001	109.435	60.612–158.259	0.258	<0.001
^d^GWW (mmHg%)	47.282	32.686–61.878	0.407	<0.001	27.003	9.078–44.928	0.232	0.003

*ARB, angiotensin receptor blockers; ACEI, angiotensin converting enzyme inhibitors; baPWV, brachial-ankle pulse wave velocity; BMI, body mass index; CCB, Calcium channel blockers; DBP, diastolic blood pressure; HR, heart rate; LVH, left ventricular hypertrophy; SBP, systolic blood pressure; UCG, ultrasound cardiogram; baPWV, brachial-ankle pulse wave velocity; CI, cardiac index; FS, fractional shortening; GCW, global constructed work; GLS, global longitude strain; GWE, global work efficiency; GWI, global work index; GWW, global wasted work; IVST, interventricular septal wall thickness; LAVI, left atrial volume index; LVEDD, left ventricular end-diastolic dimension; LVESD, left ventricular end-systolic dimension; LVEF, left ventricular ejection fraction; LVMI, left ventricular mass index; PWT, posterior wall thickness; SV, stroke volume. In multivariable linear regression analysis*.

a *Adjusted sex, hypertension, SV, LAVI, E/e' Septum, LVMI, GLS. Cumulative R^2^ = 0.553, SEE = 220.492, P < 0.001*.

b *Adjusted age, hypertension, CI, E/e' Septum, LVMI, GLS,antihypertension medication use. Cumulative R^2^ = 0.318, SEE = 2.739, P < 0.001*.

c *Adjusted sex, hypertension, SV, LAVI, E/e' Septum, LVMI, GLS. Cumulative R^2^ = 0.566, SEE = 234.113, P < 0.001*.

d *Adjusted age, hypertension, CI, SV, E/e' Septum, LVMI, GLS, antihypertension medication use. Cumulative R^2^ = 0.280, SEE = 82.435, P < 0.001*.

**Figure 3:**
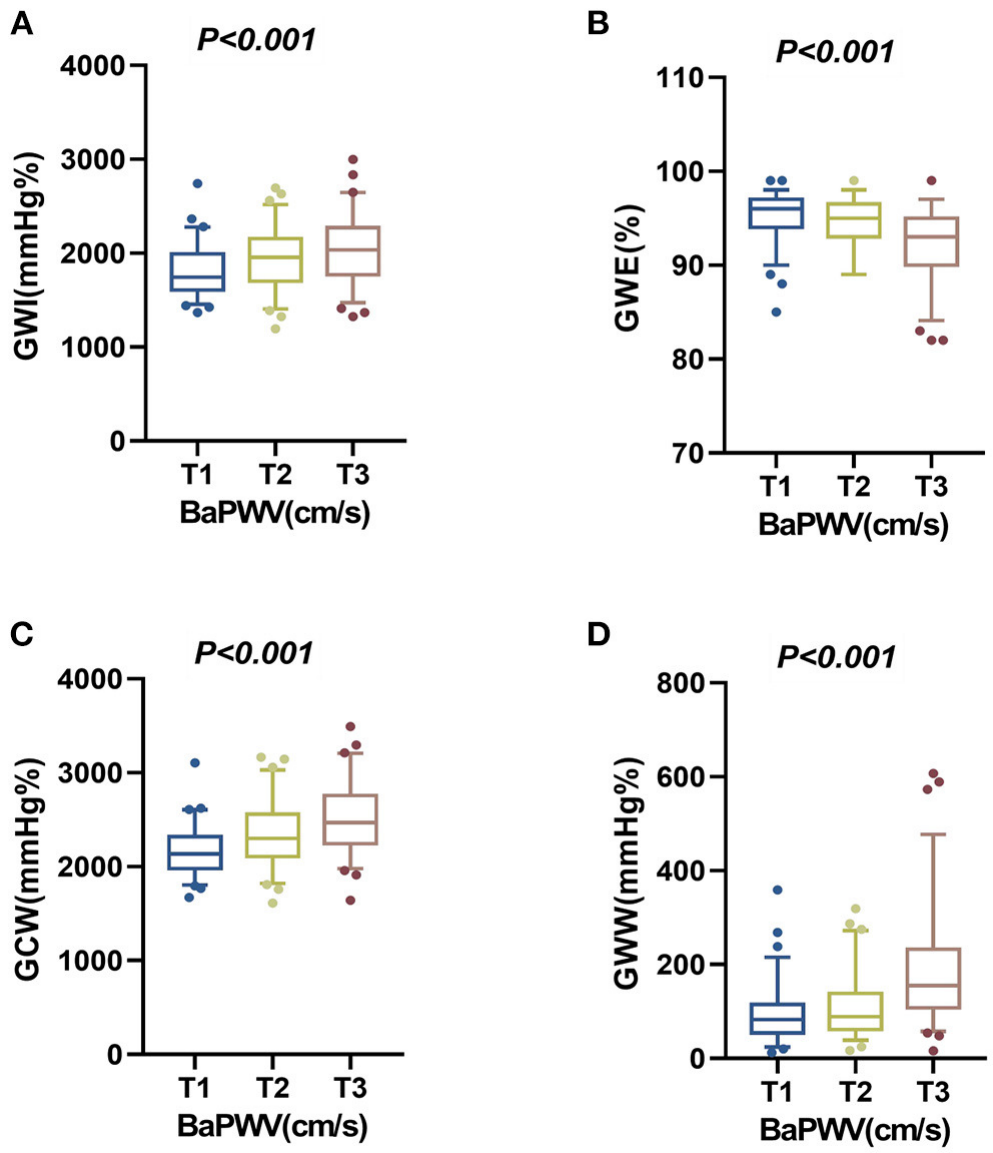
The distributions of the four components of MW among the baPWV tertile groups in the overall population. **(A)** The distribution of GWI in the baPWV tertile groups. **(B)** The distribution of GWE in the baPWV tertile groups. **(C)** The distribution of GCW in the baPWV tertile groups. **(D)** The distribution of GWW in the baPWV tertile groups. baPWV, brachial-ankle pulse wave velocity; GCW, global constructed work; GWE, global work efficiency; GWI, global work index; GWW, global wasted work; MW, myocardial work.

In multivariable linear regression analysis, as shown in Table 3, after being adjusted for the statistically significant variables in univariate analysis, higher baPWV was still an independent predictor of elevated GWI, GCW, and GWW, and poor GWE. A tertile increase in baPWV was associated with a 72.933 mmHg% increase in GWI, a 109.435 mmHg% increase in GCW, a 27.003 mmHg% increase in GWW, and a 0.738% decrease in GWE, respectively. The distributions of GWI, GWE, GCW, and GWW among the baPWV tertile groups were shown in Figures 3A–D.

**Figure 4:**
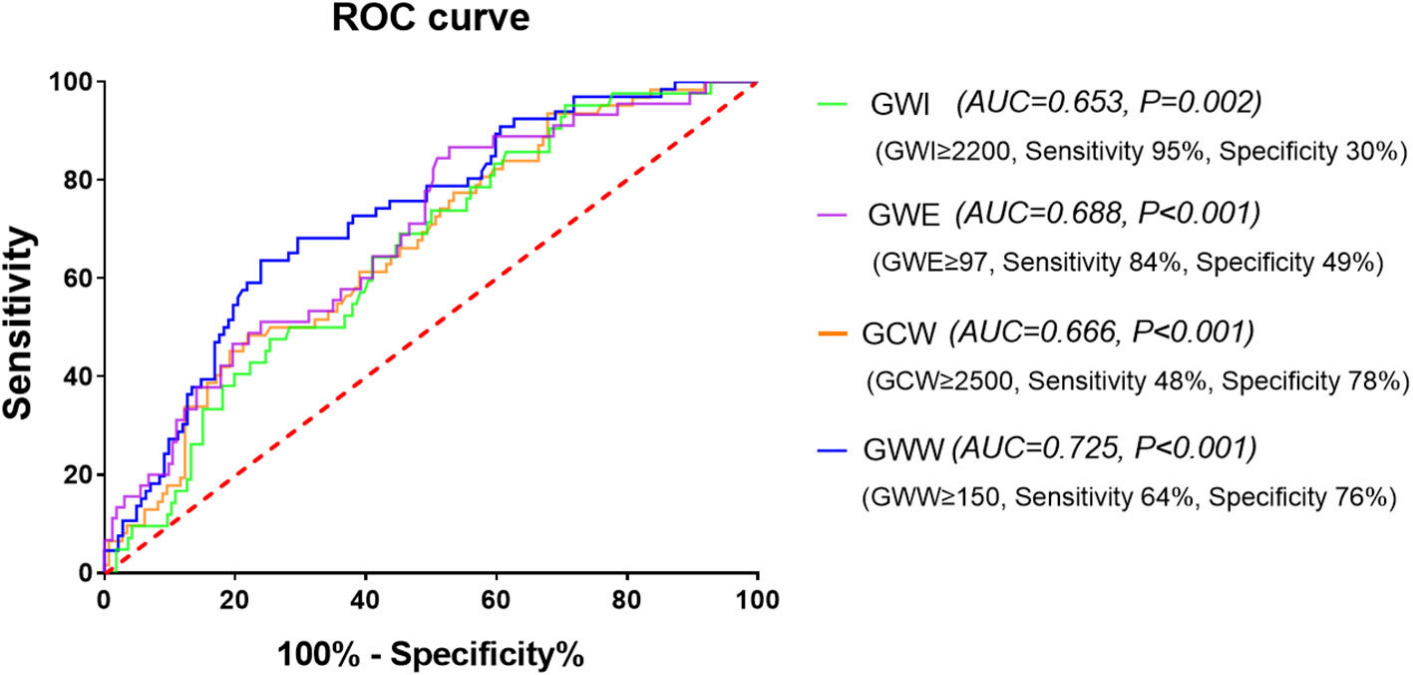
The receiver operating characteristic (ROC) curve of baPWV for predicting the four abnormal components of MW. baPWV, brachial-ankle pulse wave velocity; GCW, global constructed work; GWE, global work efficiency; GWI, global work index; GWW, global wasted work; MW, myocardial work.

The area under curve (AUC) of baPWV for predicting elevated GWI, GCW, and GWW and decreased GWE were 0.653, 0.666, 0.725, and 0.688, respectively (all *p* < 0.05). As shown in Figure 4, the cut-off values were 1,340 cm/s (sensitivity 95%, specificity 30%), 1,670 cm/s (sensitivity 48%, specificity 78%), 1,579 cm/s (sensitivity 64%, specificity 76%), and 1,544 cm/s (sensitivity 84%, specificity 49%), respectively.

## Discussion

This study showed that, in a population including both non-hypertensive and hypertensive participants with normal LVEF, baPWV was independently associated with all the four components of MW by non-invasive left ventricular pressure-strain, suggesting a close correlation between arterial stiffness and myocardial oxygen consumption. Importantly, to our knowledge, this is the first study to identify the association between arterial stiffness and MW by non-invasive left ventricular pressure-strain.

Hemodynamic interactions between the left ventricular and aorta have been considered to be a key determining factor of cardiovascular performance (22). Arterial stiffness expressed as baPWV is a strong predictor of future cardiovascular events and mortality (23). Several studies have reported the association of baPWV and LV diastolic and systolic function. Yambe et al. showed that baPWV was correlated with E/A ratio independently of other clinical variables in 147 hypertensive patients (24). Kim et al. investigated 155 apparently healthy subjects and showed a significant positive association between baPWV and the degree of E/e' ratio (25). A study by Hwang et al. demonstrated that baPWV contributed to impaired GLS and rotation of the regional myocardium in hypertensive patients with preserved ejection fraction (26). In addition, Kim et al. studied 248 subjects without structural heart problems, suggesting that baPWV was independently associated with left ventricular GLS (27). Our study is extending the association of baPWV with MW. In any given contractile state of the LV, MW has been shown to reflect the myocardial oxygen metabolism and eventually the LV function (28). We explored not only four components of MW but also other indices of left ventricular systolic function: such as, LVEF, FS, and GLS. Among these left ventricular systolic indices, all four MW components including GWI, GWE, GCW, and GWW were able to differentiate individuals with different baPWV tertiles, while LVEF and FS were not.

MW is an index of the myocardium work performance as it ejects blood during systole. Non-invasive MW and its components have been studied in several cardiac conditions, including cardiac dyssynchrony (6, 29), heart failure (30), coronary heart disease (31), hypertension (32), *etc*. It has been demonstrated that MW is a more sensitive and promising index for LV performance compared to EF and GLS. Loncaric et al. studied 170 hypertensive patients and showed that MW index increased while EF remained unaffected and GLS showed a decrease in acute pressure overload; however, in patients with chronic pressure overload, MW index was lower when myocardial remodeling and hypertrophy appeared (32). Edwards et al. studied 115 subjects referred for coronary angiography and suggested that GWI was superior to GLS to predict significant coronary artery disease in patients with no regional wall motion abnormalities and normal EF (31).

MW is considered as a less load-dependent tool for LV function evaluation as it incorporates the LV afterload. However, the protocol of this method does not include the afterload that comes from arterial stiffness (4). In aortic stiffness, reflection waves come earlier into the systole and augment the afterload, which further affect LV contraction (33). BaPWV is a good surrogate of arterial stiffness. With this study, we showed a significant association between increase arterial load and MW indices in non-hypertensive and hypertensive patients with normal LVEF. In univariate and multivariate analyses, increasing baPWV predicted higher GWI, GCW, and GWW and lower GWE, indicating an increase in myocardia oxygen consumption and a worse use of cardiac energy for the ejection of blood by the LV to the aorta. As GWE is derived from the percentage ratio of constructive work to the sum of constructive work and wasted work, the decrease of GWE implies that the increase of GWW is even more significant than that of GCW in the study population. If arterial stiffness was reduced, it might improve the efficiency of MW and presumably lower myocardial oxygen demands. Such effects are desired in this mixed normal and hypertensive population. Further studies are needed to investigate whether interventions to reduce arterial stiffness would be beneficial for the MW. The association between baPWV and MW also suggest that the protocol of MW should further involve the afterload generated by the arterial tree, as the pressure-strain loop represents the ventricular-arterial coupling, which is continuously changing to match ventricular end-systolic and arterial elastances.

This study is not without limitations. the first limitation was the short age range and small sample size. This would render the drawn conclusions less reliable. However, we sought to exclude all patients with comorbidities that might affect LV function like diabetes, ischemic heart disease, which commonly coexist with hypertension, and hence the effect encountered would be solely attributed to hypertension. Second, we could not test simultaneously systolic blood pressure and baPWV because of their high level of collinearity. Third, the study was based in a single center population. A causal relationship between baPWV and MW could not be ascertained because of the cross-sectional design. Fourth, although we have excluded chronic kidney disease and valve heart disease in this study, there are still some pitfalls when evaluating baPWV, including arteriosclerosis obliterans, arterial calcification in the lower limb and arterial stenosis in the right upper-limb (34). However, An ABI of 1 or greater for all study populations have largely avoided these pitfalls.

In summary, our findings indicate that the baPWV is significantly associated with MW by a non-invasive left ventricular pressure-strain method in a mixed population of non-hypertensive and hypertensive individuals. This confirms the role of baPWV as a preclinical marker of target organ damage extending its significant impact on MW. This association is independent of major confounders, such as LVH, left ventricular diastolic dysfunction, and GLS. Individuals with increased baPWV could be therefore identified as a risk factor for development of subsequent heart failure.

## Data Availability Statement

The original contributions presented in the study are included in the article/Supplementary Material, further inquiries can be directed to the corresponding authors.

## Ethics Statement

This study was registered in clinicaltrials.gov (approval No. NCT04573257). It was conducted in accordance with the Declaration of Helsinki and approved by the Ethics Committee of the First Affiliated Hospital of Chongqing Medical University (approval No. 2020-606). Informed consent was obtained from all patients. The patients/participants provided their written informed consent to participate in this study.

## Author Contributions

QDu, DZ, QDo, KL, YY, and LY performed the research and analyzed data. QDu and PG wrote the manuscript. QDu and SQ designed the study and critically reviewed the manuscript. All authors reviewed the manuscript. All authors contributed to the article and approved the submitted version.

## Funding

This study was supported by Chongqing Science and Technology Commission, Key Project, (2019 ZLXM003); Chongqing Science and Technology Commission, Youth Project, (2018QNXM024).

## Conflict of Interest

The authors declare that the research was conducted in the absence of any commercial or financial relationships that could be construed as a potential conflict of interest.

## Publisher's Note

All claims expressed in this article are solely those of the authors and do not necessarily represent those of their affiliated organizations, or those of the publisher, the editors and the reviewers. Any product that may be evaluated in this article, or claim that may be made by its manufacturer, is not guaranteed or endorsed by the publisher.
