# Supplementary material for: Impact of Brachial-Ankle Pulse Wave Velocity on Myocardial Work by Non-invasive Left Ventricular Pressure-Strain in Non-hypertensive and Hypertensive Patients With Preserved Left Ventricular Ejection Fraction
Source: Front Cardiovasc Med. 2022 Feb 10;9:814326. doi: 10.3389/fcvm.2022.814326 (PMC8866308; doi:10.3389/fcvm.2022.814326)
Supplement: Supplementary file 1 [file Data_Sheet_1.docx]

**Supplementary table S1.** Baseline clinical characteristic and echocardiographic data in non-hypertensive and hypertensive study participants.

| Variable | Non-hypertensive  (n = 104) | Hypertensive  (n = 104) | *P* | |
| --- | --- | --- | --- | --- |
| \| Baseline clinical data \| \| --- \| | | | | |
| Age (years) | 48.5 (39.3 - 55.0) | 50.0 (44.3 - 56.8) | 0.169 | |
| Male/Female | 46/58 | 68/36 | 0.002 | |
| SBP (mmHg) | 121.0 (113.0 - 131.0) | 144.0 (135.0 - 154.8) | < 0.001 | |
| DBP (mmHg) | 77.0 (72.0 - 82.0) | 90.5 (84.25 - 97.75) | < 0.001 | |
| HR (bpm) | 75.2 (64.6 - 84.7) | 76.6 (66.4 - 83.7) | 0.402 | |
| baPWV (cm/s) | 1379.0 (1249.3 - 1506.3) | 1615.0 (1469.0 - 1827.5) | < 0.001 | |
| BMI (kg/m^2^) | 22.8 (21.3 - 25.3) | 25.3 (23.6 - 27.5) | < 0.001 | |
| Waist (cm) | 81.5 ± 9.1 | 87.8 ± 9.5 | < 0.001 | |
| Current smoking | 19 (18.3%) | 34 (32.7%) | 0.017 | |
| Current drinking | 20 (19.2%) | 34 (32.7%) | 0.027 | |
| \| Standard echocardiographic data \| \| --- \| | | | | |
| IVST (mm) | 9.5 (9.0 - 10.0) | 11.0 (10.0 - 12.0) | < 0.001 | |
| PWT (mm) | 9.0 (9.0 - 10.0) | 11.0 (9.0 - 12.0) | < 0.001 | |
| LVEDD (mm) | 45.0 (43.0 - 47.0) | 47.0 (44.0 - 49.0) | 0.004 | |
| LVESD (mm) | 29.0 (27.0 - 31.0) | 30.0 (28.5 - 32.0) | 0.003 | |
| FS (%) | 35.0 (33.0 - 36.0) | 35.0 (32.5 - 37.0) | 0.847 | |
| LVEF (%) | 64.0 (62.0 - 66.0) | 64.0 (61.0 - 67.0) | 0.863 | |
| CI (L/min/m^2^) | 2.7 ± 0.6 | 2.8 ± 0.6 | 0.439 | |
| SV (ml) | 61.5 (51.7 - 65.2) | 63.0 (55.8 - 71.6) | 0.006 | |
| LVMI (g/m^2^) | 84.9 (78.4 - 95.8) | 103.7 (91.3 - 118.8) | < 0.001 | |
| LAVI (ml/m^2^) | 25.1 (21.3 - 31.5) | 25.1 (22.1 - 31.0) | 0.811 | |
| E/e’ Septum | 7.5 (6.1 - 9.4) | 6.1 (5.0 - 7.6) | < 0.001 | |
| Myocardial work and strain data | | | |  |
| GWI (mmHg%) | 1839.7 ± 270.9 | 2021.7 ± 348.0 | < 0.001 | |
| GWE (%) | 96.0 (94.0 - 97.0) | 94.0 (91.3 - 96.0) | < 0.001 | |
| GCW (mmHg%) | 2217.4 ± 279.9 | 2454.3 ± 371.3 | < 0.001 | |
| GWW (mmHg%) | 86.5 (57.0 - 128.0) | 128.0 (84.3 - 204.8) | < 0.001 | |
| GLS (%) | 20.4 ± 1.9 | 19.0 ± 2.1 | < 0.001 | |

BaPWV, brachial-ankle pulse wave velocity; BMI, body mass index; CI, cardiac index; DBP, diastolic blood pressure; FS, fractional shortening; GCW, global constructed work; GLS, global longitude strain; GWE, global work efficiency; GWI, global work index; GWW, global wasted work; HR, heart rate; IVST, interventricular septal wall thickness; LAVI, left atrial volume index; LVEDD, left ventricular end-diastolic dimension; LVESD, left ventricular end-systolic dimension; LVEF, left ventricular ejection fraction; LVMI, left ventricular mass index; PWT, posterior wall thickness; SBP, systolic blood pressure; SV, stroke volume.

**Supplementary table S2.** Correlation of GWI, GWE, GCW and GWW with major clinical and echocardiographic parameters in the study population.

| Variable | GWI | |  | GWE | |  | GCW | |  | GWW | |
| --- | --- | --- | --- | --- | --- | --- | --- | --- | --- | --- | --- |
|  | Coefficient | *P* |  | Coefficient | *P* |  | Coefficient | *P* |  | Coefficient | *P* |
| Age (years) | 0.112 | 0.108 |  | -0.250 | < 0.001 |  | 0.112 | 0.108 |  | 0.267 | < 0.001 |
| Sex (Male vs Female) | 0.145 | 0.036 |  | 0.091 | 0.190 |  | 0.120 | 0.084 |  | -0.058 | 0.407 |
| BMI (kg/m^2^) | -0.01 | 0.888 |  | -0.119 | 0.086 |  | -0.015 | 0.835 |  | 0.096 | 0.169 |
| Current smoking (no vs yes) | -0.073 | 0.294 |  | -0.017 | 0.805 |  | -0.093 | 0.182 |  | -0.008 | 0.905 |
| Current drinking (no vs yes) | -0.047 | 0.497 |  | -0.039 | 0.579 |  | -0.067 | 0.335 |  | -0.007 | 0.917 |
| Hypertension (no vs yes) | 0.266 | < 0.001 |  | -0.289 | < 0.001 |  | 0.321 | < 0.001 |  | 0.307 | < 0.001 |
| LVEF (%) | 0.062 | 0.378 |  | 0.010 | 0.888 |  | 0.018 | 0.798 |  | -0.003 | 0.971 |
| CI (L/min/m^2^) | -0.039 | 0.581 |  | -0.166 | 0.017 |  | 0.133 | 0.056 |  | 0.194 | 0.005 |
| SV (ml) | 0.096 | 0.167 |  | -0.106 | 0.130 |  | 0.166 | 0.017 |  | 0.131 | 0.060 |
| LAVI (ml/m^2^) | 0.383 | < 0.001 |  | -0.027 | 0.701 |  | 0.349 | < 0.001 |  | 0.083 | 0.236 |
| E/e’ Septum | 0.374 | < 0.001 |  | -0.253 | < 0.001 |  | 0.392 | < 0.001 |  | 0.317 | < 0.001 |
| LVMI (g/m^2^) | 0.387 | < 0.001 |  | -0.343 | < 0.001 |  | 0.338 | < 0.001 |  | 0.375 | < 0.001 |
| GLS (%) | 0.423 | < 0.001 |  | 0.471 | < 0.001 |  | 0.375 | < 0.001 |  | -0.363 | < 0.001 |
| BaPWV (cm/s) | 0.338 | < 0.001 |  | -0.383 | < 0.001 |  | 0.417 | < 0.001 |  | 0.449 | < 0.001 |
| baPWV tertiles | 0.316 | < 0.001 |  | -0.378 | < 0.001 |  | 0.387 | < 0.001 |  | 0.437 | < 0.001 |

BaPWV, brachial-ankle pulse wave velocity; BMI, body mass index; CI, cardiac index; GCW, global constructed work; GLS, global longitude strain; GWE, global work efficiency; GWI, global work index; GWW, global wasted work; LAVI, left atrial volume index; LVEF, left ventricular ejection fraction; LVMI, left ventricular mass index; SV, stroke volume.

**Supplementary Fig S1.** Correlation between baPWV and 4 components of myocardial work in both non-hypertensive and hypertensive subgroups.


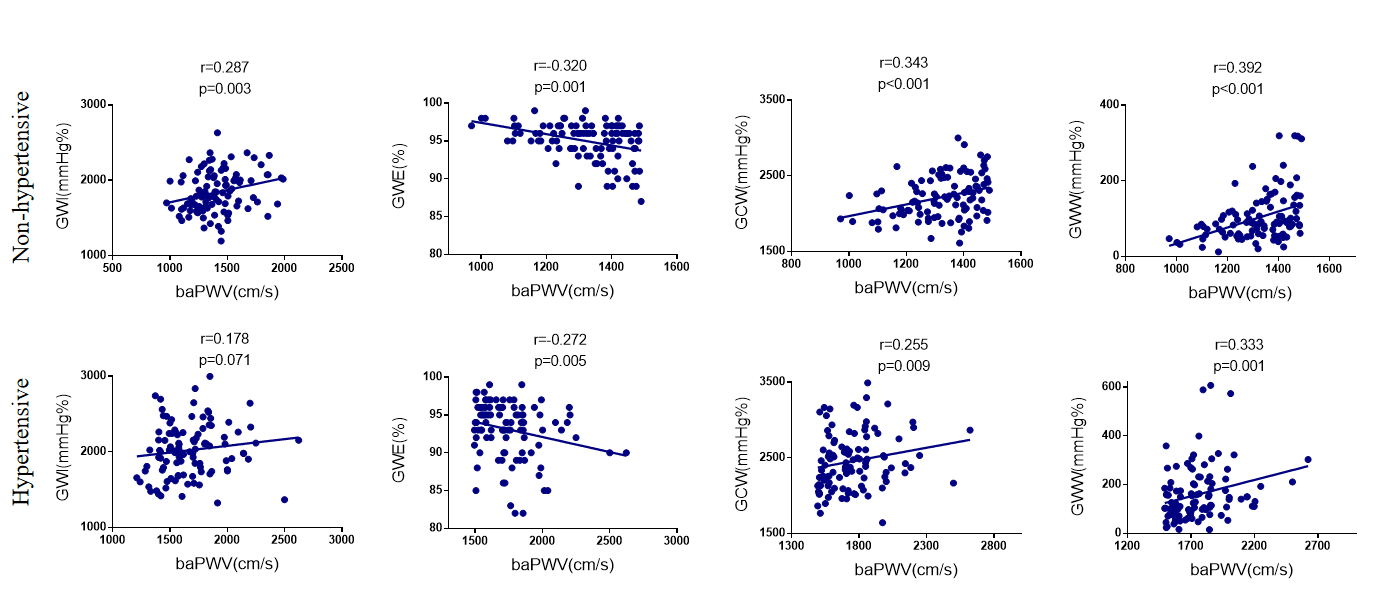


BaPWV, brachial-ankle pulse wave velocity; GCW, global constructed work; GWE, global work efficiency; GWI, global work index; GWW, global wasted work.
